# Supplementary material for: Automated Service Height Fault Detection Using Computer Vision and Machine Learning for Badminton Matches
Source: Sensors (Basel). 2023 Dec 11;23(24):9759. doi: 10.3390/s23249759 (PMC10747833; doi:10.3390/s23249759)
Supplement: Supplementary file 1 [file sensors-23-09759-s001.zip › supplementary information.pdf]

Supplementary information

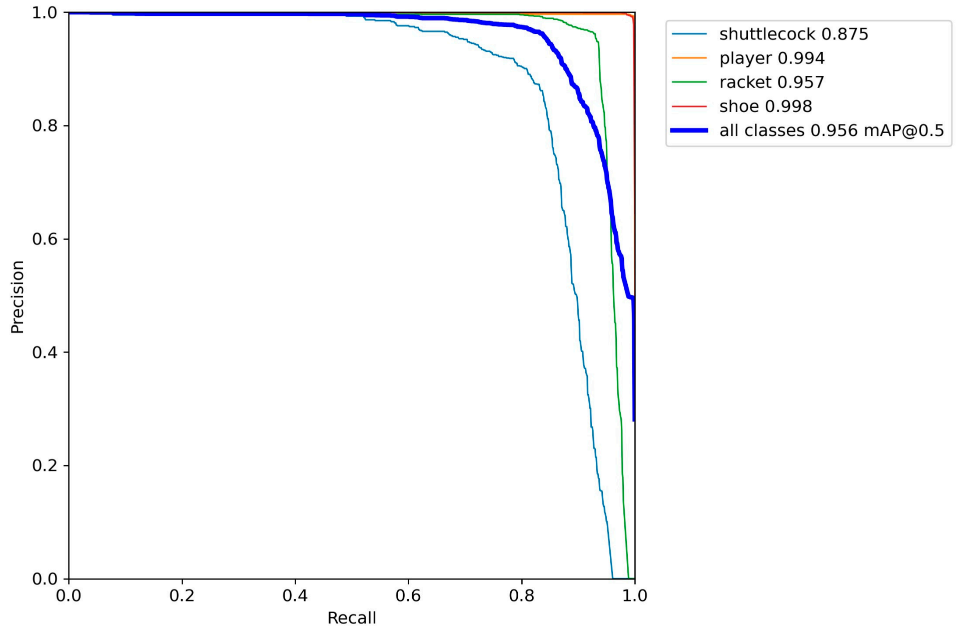

Figure S1. Precision-Recall curve of the trained model using untrained dataset
